# Assessing Sensorimotor Synchronisation in Toddlers Using the Lookit Online Experiment Platform and Automated Movement Extraction

**DOI:** 10.3389/fpsyg.2022.897230

**Published:** 2022-06-30

**Authors:** Sinead Rocha, Caspar Addyman

**Affiliations:** ^1^School of Psychology and Sport Science, Anglia Ruskin University, Cambridge, United Kingdom; ^2^Department of Psychology, University of Cambridge, Cambridge, United Kingdom; ^3^Department of Psychology, Goldsmiths, University of London, London, United Kingdom

**Keywords:** sensorimotor synchronisation, infancy, development, machine learning, OpenPose, Lookit, automated movement analysis

## Abstract

Adapting gross motor movement to match the tempo of auditory rhythmic stimulation (sensorimotor synchronisation; SMS) is a complex skill with a long developmental trajectory. Drumming tasks have previously been employed with infants and young children to measure the emergence of rhythmic entrainment, and may provide a tool for identification of those with atypical rhythm perception and production. Here we describe a new protocol for measuring infant rhythmic movement that can be employed at scale. In the current study, 50 two-year-olds drummed along with the audiovisual presentation of four steady rhythms, using videos of isochronous drumming at 400, 500, 600, and 700 ms IOI, and provided their spontaneous motor tempo (SMT) by drumming in silence. Toddlers’ drumming is observed from video recordings made in participants’ own homes, obtained *via* the Lookit platform for online infant studies. We use OpenPose deep-learning model to generate wireframe estimates of hand and body location for each video. The vertical displacement of the hand was extracted, and the power and frequency of infants’ rhythmic entrainment quantified using Fast Fourier Transforms. We find evidence for age-appropriate tempo-flexibility in our sample. Our results demonstrate the feasibility of a fully digital approach to measuring rhythmic entrainment from within the participant’s home, from early in development.

## Introduction

Rhythmic timing underlies a broad set of human behaviours, including music and dance. Critically, the emerging ability to produce an internally generated rhythm (spontaneous motor tempo; SMT), and adapt one’s movement to match an external stimulus (sensorimotor synchronisation; SMS), is related to success in language acquisition. Poor SMS is related to language difficulties in typically developing pre-schoolers (Carr et al., 2014; Politimou et al., 2019; Rios-Lopez et al., 2019), and across language disorders, including dyslexia (Thomson and Goswami, 2008; Lee et al., 2015; Persici et al., 2019), developmental language disorder (DLD; Corriveau and Goswami, 2009; Cumming et al., 2015), and speech impediments (Olander et al., 2010; Falk et al., 2015). Sensorimotor synchronisation is suggested as a useful tool for identifying those who may struggle with language, which is suitable from infancy and through the lifespan (Ladányi et al., 2020).

Since Fraisse (1982), laboratory measures of SMT in adulthood are often measured *via* tapping paradigms, where discreet intervals are produced by the vertical displacement of the index finger onto a keypad or equivalent surface. Whilst in adulthood the self-produced rate of tapping is stable within-subjects over short periods of time (Vanneste et al., 2001), across the lifespan SMT is known to change, with children’s tapping significantly faster than adult’s (McAuley et al., 2006). Adult SMT is in the range of 630 ms inter-onset-interval (IOI; McAuley et al., 2006). In very early childhood, SMT measured *via* tapping has been demonstrated as fast as 400–450 ms (IOI; Bobin-Bègue and Provasi, 2008). Whilst tapping dominates the SMT literature, and adult SMS studies, across early childhood, this difficult fine-motor task is not always the most appropriate. Particularly in determining infant ability to synchronize with external stimuli, different research groups have used a variety of tasks to facilitate synchrony within populations with poor fine motor skills.

Seminal studies into infant movement to music have simply allowed infants to move freely to auditory stimuli (Zentner and Eerola, 2010; Fujii et al., 2014). However, such scenarios do not provide auditory/haptic feedback equivalent to the tapping measures used in adulthood. Other infant paradigms used small hand-held instruments such as bells (Rocha and Mareschal, 2017). The closest experimental paradigm to tapping involves whole-hand drumming. From 5 months-of-age, infants can produce their own SMT *via* drumming (Rocha et al., 2021b) with the tempo and regularity of their drumming increasing over the first 2 years of life. Whilst infants cannot reliably synchronise their movements to music, a longitudinal investigation of infant drumming to nursery rhymes of different tempi suggests that by 11-months-of-age infants are beginning to shift away from their SMT to better match the rate of the song (Rocha et al., 2021a). Studies of toddlers evidence good tempo adaptation in older infants, when drumming along with a human and non-human partner (Kirschner and Tomasello, 2009; Yu and Myowa, 2021). In contrast to whole-body free movement analysis, constraints imposed in a drumming task allow more direct comparison of SMS over age, with a common effector and motion as is commonly used in adult tapping studies. As drumming can be used across contexts, with minimal apparatus and instruction, and from 5-months-of-age with no upper limit, we suggest that this could be a candidate marker of SMS that could be used at scale to detect early individual differences. Whilst there is a strong movement toward identification of risk of language disorders using neural markers, e.g., (Attaheri et al., 2022) an accessible behavioural assessment of rhythmic skill would have multiple practical advantages in identifying children at risk.

In the current study we test the feasibility of measuring SMS in the child’s own home, using asynchronous data collection methods, and largely automated data processing. If viable, such a technique will allow for large scale data collection. Our approach is focussed on creating an open source tool to evaluate rhythm in developing populations using a task that is low cost, easy to administer, and easy to adapt for research and clinical needs. Here we describe the implementation of our paradigm on a group of 2-year-olds, a notoriously difficult age to test, and document the successes and failures of our approach. We first ask whether we can detect the rate of drumming from home-video footage. We then characterise toddler Spontaneous Motor Tempo, and ask whether toddlers of this age show signs of successful SMS at a group level.

## Materials and Methods

### Participants

The initial sample included 68 infants who completed the online drumming task. These include 39 female, 28 male and one gender not specified. Their mean age was 816.2 ± 94.1 days. A further 24 participants (12 female) were excluded because they did not complete the task (22) or withdrew (2). Participants were recruited through the Lookit website and *via* the experimenters’ research networks. Ethical approval for the study was obtained from Psychology Ethics Committee at Goldsmiths, University of London.

### Design

**FIGURE 1:**
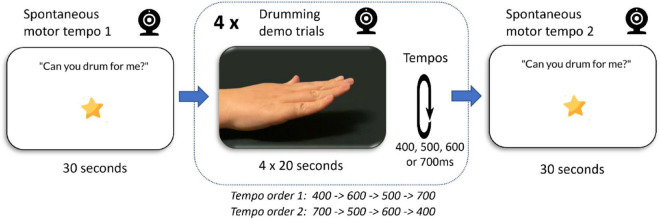
Schematic of the data collection trials. Using the Lookit platform, children saw six short videos and their responses were video recorded. First and last trial lasted 20 s and captured infants own spontaneous drumming. Trials 2–5 each demonstrated drumming at four different tempos (an inter-stimulus intervals of 400, 500, 600, or 700 ms per beat).

The study used a mixed design with all participants completing the same set of six video recorded trials, with counterbalanced order of target inter-stimulus intervals. The first and sixth trials were to designed measure spontaneous motor tempo. The middle four trials each demonstrated drumming at a different interonset interval (IOI) ranging from 400 to 700 ms in steps of 100 ms, chosen to capture the possible range of SMT over childhood. In order one these were presented in the sequence (400, 600, 500, and 700), and in order two (700, 500, 600, and 400; See Figure 1).

### Materials

The demographic and video data were collected on the Lookit online child lab website (Scott and Schulz, 2017). The Lookit website^1^ managed the sign up of participants and collection of demographic details (date of birth, sex, race, geographic location, number of children in the family, languages, parent education level, household income, number of children’s books at home). It presented informed consent and data-sharing agreements for caregivers. During the data collection phase Lookit presented the stimulus and reward videos, created by the experimenters. For both Spontaneous Motor Tempo trials, the same 20-s-long silent video was used. It displayed written prompts “Can you drum for me?,” “What sound does it make when you drum?,” for the caregiver to read aloud to the infant. In the experimental trials, 20 s videos showed a woman’s hand tapping out a steady beat on a flat surface at an interval of 400, 500, 600, or 700 ms. All the materials are available online at https://github.com/InfantLab/little-drummers.

### Procedure

Data collection took place in participants own homes with caregivers following online instructions to run the experiment using their own personal computers. Prior to the study, caregivers created an account on the Lookit website and provided basic demographic details. At a time of their choosing they recorded verbal consent to their participation and followed instructions to position their child in view of their webcam. They were asked not to have the child on their lap and make sure that child’s hands were visible in the shot, though compliance with these instructions could not be ensured. The camera view was shown on screen to help with positioning.

When child and caregiver were in position the six trials began. The trials progressed automatically but caregivers could pause the study by pressing the spacebar. If a trial was paused, it could be restarted or the caregiver could choose to end the study early if the child became too fussy. To capture children’s spontaneous motor tempo, the first and last trial provided no tempo information but showed onscreen prompts for the caregiver to encourage drumming. Parents were instructed not to demonstrate drumming themselves. The experiment software randomly assigned participants to one of two order conditions which determined the sequence of Trials 2–5. In each of these trials a 20 s long video of a woman’s hand drumming on table was presented. The videos were accompanied by an onscreen caption “Adults, in this video please say ‘Can you drum along’?”. Each trial was followed by a 5 s “reward” video. Following data collection, caregivers were asked to confirm their child’s date of birth and specify a level of data sharing (Public, Scientific, and Private). Finally, a debrief explained the experiment and thanked them for their participation. A video walkthrough of the experiment can be found online at https://github.com/InfantLab/little-drummers#experiment-walkthrough.

### Data Analysis

**FIGURE 2:**
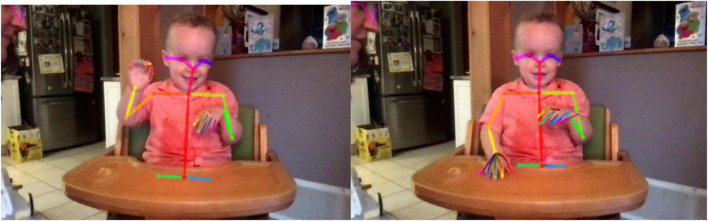
A toddler filmed drumming at home using the Lookit platform. Parents supervise the study following instructions on laptop screen. Wireframe overlay created using OpenPose (Cao et al., 2021).

Our novel approach to coding infant rhythmic behaviour uses the OpenPose software for markerless motion tracking (Cao et al., 2021). OpenPose is a deep learning model that has been trained to identify multiple human figures in images and video that is widely used in research settings (e.g., Fujiwara and Yokomitsu, 2021; Kim et al., 2021; Zeng and Chen, 2021). When presented with a video it analyses each frame independently, labelling all people present. For each identified person, it can tag up to 25 key points on the body (depending on visibility) and has an optional hand-model that identifies up to 21 key points per hand. Each identified marker is given as x and y coordinates within the frame (see Figure 2). OpenPose also has the ability to label face markers but this was not used in the current project. OpenPose is an open source project that is free to use in non-commercial applications (For further information, see https://github.com/CMU-Perceptual-Computing-Lab/openpose).

**FIGURE 3:**
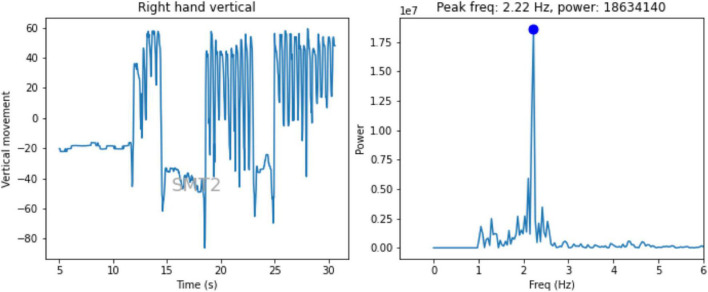
**(A)** The graph shows the vertical movement of the toddler’s right hand using averaged OpenPose data over their second spontaneous motor tempo trial, which contained three bursts of drumming. **(B)** The graph shows the power spectrum derived using fast fourier transform of the data, revealing a spontaneous motor frequency of 2.22 Hz.

Using the keypoint data generated by OpenPose, the vertical displacement of hand can then be extracted and the power and frequency of infants rhythmic entrainment can be measured using Fast Fourier Transforms (see Figure 3). However, to make use of the raw data generated by OpenPose, a substantial amount of additional data processing is required. In this section, we briefly walk-through the steps involved in data transformation, cleaning and analysis.

All analysis was performed in Python using the Jupyter notebooks interface which creates an annotated analysis script, allowing for direct reproduction of all analysis steps. A general toolkit for performing these steps and a short tutorial are freely available (open source) at https://github.com/InfantLab/VASC. The specific versions of libraries, scripts and their output for this dataset are found at https://github.com/InfantLab/little-drummers.

#### Step 1: Motion Capture Video Conversion

Lookit provides video for each individual trial as a separate file with a unique identifier per child and condition. We downloaded all videos for all children and all conditions. The Step 1 script then passed each video to OpenPose. It processes videos frame-by-frame outputting a single structured data file per frame (JSON format), containing all key point information (screen X- and Y- coordinates and a percentage confidence score per key point). OpenPose processed all 402 videos in our dataset, producing approximately 250,000 JSON files. Next the script parsed the outputted JSON to extract and combine all data into a single multi-dimensional NumPy array. We save this in a compressed format to pass to Step 2.

#### Step 2: Data Cleaning and Collation

The OpenPose software has limitations so a considerable amount of data cleaning is required. For each video the experimenters had to manual check the data generated by OpenPose and make sure it correctly identified the drumming infant. The biggest problem is that OpenPose operates on a per frame basis and so can have inconsistent labelling between frames. For example, it may label infant and caregiver as person 0 and person 1 in one frame but as person 1 and person 0 in the next. Additionally, there may be additional people who temporarily enter the field of view or OpenPose can include false negatives (failure to label person) and false positives (labelling background scenery as a “ghost” person). The step 2 script provides visual inspection tools for manual corrections. It allows the experimenter to see plots of the average locations of the figures in the video across the whole time-series. Mislabelling shows up as large jumps and cross-overs in the plots. The user can select the affected frame and relabel the data and remove erroneous false positives. To speed up this process a set of simple automatic algorithms to relabel the figures consistently were created. These operate by comparing each frame to the one previous and matching the figure labels by location or by figure size. More details can be found in the online tutorial. The output of this process is a multidimensional time-series of consistently labelled body and hand points for each infant in each trial. We saved these as multi-index Pandas dataframes to pass to Step 3b.

#### Step 3a: Manual Tagging of Drumming Trials

To identify videos without drumming, experimenters watched all videos and manually coded infant behaviour. For each trial we recorded whether the infants hands were visible, whether they drummed with either left hand, right hand or both, and whether there was any interference from the caregiver. Trials were retained for analysis if infants took at least four consecutive strikes (<2,000 ms between hits) of the surface in front of them, and discarded if an adult moved the infants’ hand themselves. 18 infants demonstrated no drumming, from the remaining 50 participant drumming was seen in a total of 208 trials (Mean = 4.16 per infant). These were labelled in a spreadsheet that was read by Step 3b.

#### Step 3b: Extracting Rhythmic Hand Data With Fast Fourier Transforms

For each trial we first linearly interpolated any missing data in the time series of marker points. Across the 208 trails selected in Step 3a, this affected less than 1% of the data. Then we found the average location of left and right hands by creating weighted sums of the hand and wrist X- and Y-coordinates. We use this derived y-coordinate as a measure of the vertical movement of the drumming hand over time. Next, we subtracted the mean vertical displacement in a given trial from each of the time-series to give a measure of movement. This data was then transformed into a power spectrum using the discrete Fourier transform routines in SciPy (Virtanen et al., 2020). To filter out larger, non-rhythmical movements we cut off the power spectrum below 1 Hz. The maximum power was found and the corresponding frequency saved as the tempo for that trial for each hand. The infants “best” hand (i.e., with an FFT with the highest power) is used in further analyses. See Figures 3A,B for example of vertical movement and corresponding power spectrum.

## Results

A total of 68 infants completed the experiment. Of these, 18 demonstrated no visible drumming and are excluded from further analysis. This included 14 who were partially off-camera, and a further four who presented no drumming in any trial. The remaining 50 infants provided drumming data in 208 different trials averaging 1.12 SMT trials each and 3.04 drumming trials across the different conditions. For each trial, drumming frequency was recorded for subsequent analysis.

### Rate of Drumming

**FIGURE 4:**
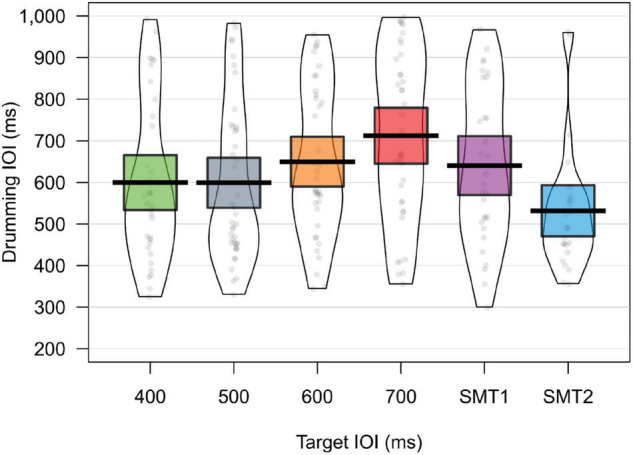
Figure shows violin plots and jittered raw data of the rate of infant drumming in each experimental condition. The Mean is signified by a bold line, and the 95% CI is shaded. Whilst infant drumming does not directly correspond to the rate of presentation, infants appear to be adapting their rate of drumming to be slower in the slow trials (600 and 700 ms IOI).

The median IOI of infant drumming in silence (SMT) trial 1 was 608 ms, decreasing to 491 ms at trial 6. Infant drumming during stimulation appears to show some tempo flexibility (i.e., slower drumming to longer IOIs), see Table 1 and Figure 4.

**TABLE 1:** Inter-onset-interval (IOI) of infant drumming derived from FFT.

Target IOI (ms)	N	Mean	SD	Median	SE
700	38	712.208	204.097	695.797	33.109
600	38	649.802	182.196	596.068	29.556
500	41	599.066	190.883	523.657	29.811
400	35	599.418	192.16	549.679	32.481
SMT1	30	640.453	189.621	607.89	34.62
SMT2	26	531.843	152.001	491.127	29.81

*N reflects number of infants that drummed in each trial.*

In order to test whether infants were indeed drumming at different rates across the different IOI trial types, a linear mixed effects model with a random slope on participant was conducted in RStudio Team (2020.09.01), RStudio (2021.09.01) (RStudio Team, 2020), with the specification “rate of drumming ∼ trial type + (1| participant)”. The slowest, 700 ms condition was taken as the basecase. An ANOVA using Satterthwaite’s method reveals a highly significant main effect of trial IOI (*F* = 3.573, *p* = 0.004). Full results are shown in Table 2. *Post-hoc* tests show that all tempi except 600 ms and SMT1 elicited significantly faster drumming than in the 700 ms trial.

**TABLE 2:** Table of coefficients for linear mixed effect models.

	Rate of drumming			Tempo mismatch		
*Predictors*	*Estimates*	*CI*	*p*	*Estimates*	*CI*	*p*
(Intercept)	713.88	654.31 to 773.45	**<0.001**	175.84	132.17 to 219.50	**<0.001**
600 ms	−63.87	−141.97 to 14.23	0.108	−24.98	−84.19 to 34.23	0.406
500 ms	−114.42	−190.52 to −38.31	**0.003**	−19.26	−77.13 to 38.61	0.512
400 ms	−110.05	−190.17 to −29.94	**0.007**	35.73	−24.94 to 96.40	0.246
SMT 1	−72.96	−156.93 to 11.00	0.088			
SMT 2	−168.01	−255.25 to −80.78	**<0.001**			
**Random effects**						
σ^2^	29,036.09			16,769.25		
τ_00_	6,176.53 _Participant_			1,850.26 _Participant_		
ICC	0.18			0.10		
N	50 _Participant_			49 _Participant_		
Observations	208			152		
Marginal R^2^/Conditional R^2^	0.068/0.232			0.028/0.125		

*Bold values indicate significant values at p < 0.05.*

### Tempo Mismatch

**FIGURE 5:**
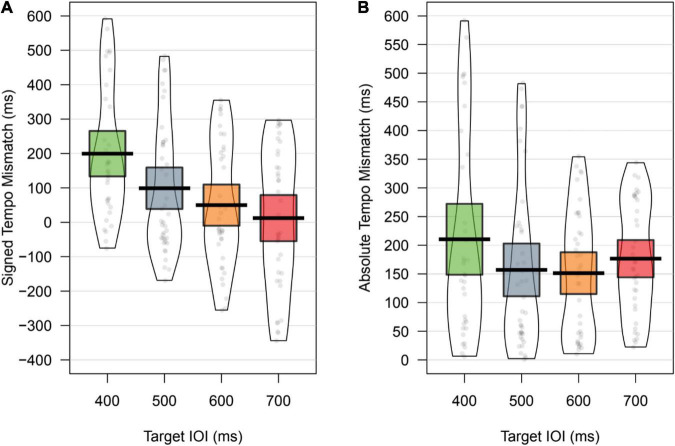
Panel **(A)** depicts the tempo mismatch of infant drumming (IOI of drumming – Target IOI). Positive numbers reflect drumming slower than target, and negative numbers reflect drumming faster than target. Values close to zero reflect accurate drumming. Panel **(B)** depicts the absolute (non-signed) tempo mismatch. The Mean is signified by a bold line, and the 95% CI is shaded.

In order to quantify how accurately infants were tempo-matching during the different tempo trials, we calculated a tempo mismatch score as the rate of infant drumming minus the target IOI. Positive mismatch values therefore reflect infants drumming slower than the target IOI, and negative values reflect faster than target drumming. Tempo mismatch is plotted in Figure 5A.

For further analysis, the absolute (i.e., non-signed) tempo mismatch is taken as the dependent variable, see Figure 5B. Descriptive statistics are shown in Table 3. The mismatch between infant drumming and the target IOI was approximately 150–200 ms, across the four target tempi.

**TABLE 3:** Tempo mismatch of infant drumming at each target IOI.

Target IOI (ms)	N	Mean	SD	Median	SE
700	38	176.558	98.963	165.026	16.054
600	38	151.344	110.538	153.469	17.932
500	41	157.076	145.624	109.394	22.743
400	35	210.369	179.738	149.679	30.381

If infants are not tempo-matching, we would expect to see higher “accuracy,” or lower tempo-mismatch, in the trials with a target tempo closer to their SMT. To test for this pattern a further linear mixed model with a random slope on participant was conducted, with the specification “tempo mismatch ∼ trial IOI + (1| participant)”. Data provided in the SMT conditions were not included, as there was no target for infants to match. We do not find a main effect of trial IOI (*F* = 1.605, *p* = 0.192) nor any *post hoc* differences, suggesting that infants performed similarly across conditions (all *p* n.s., see Table 2).

Finally, as infant SMT in trial SMT1 was slower than predicted for this age group, manual video coding was used to determine if SMT was related to the number of hits performed in each trial. Descriptive statistics for all trials are presented in Table 4.

**TABLE 4:** Number of drum hits made by infants in each trial type.

Target IOI (ms)	N	Mean	SD	Median	Minimum	Maximum
400	38	23.171	12.215	22.000	4	45
500	38	25.525	13.263	24.500	6	53
600	41	22.816	13.096	24.000	4	51
700	35	18.395	10.709	16.000	4	49
SMT1	30	16.800	7.284	17.000	4	35
SMT2	26	23.500	13.064	20.000	6	51

Infants were indeed seemingly less engaged in the SMT trials (where there was no drumming video to follow), reflected in both a lower N of infants participating in these trials, and a lower number of hits by those who did participate in SMT1. Notably, after the presentation of drumming videos, in SMT2, infants who did participate were drumming to a similar extent as during the test trials. It is therefore possible that the slower than expected SMT for this age group recorded in SMT1 is the product of infants not producing a reliable estimate due to insufficient data. If this were the case, we might expect that infants who drummed more in this trial would have a faster SMT. However, SMT1 is not significantly correlated with the number of hits produced, with evidence for the null hypothesis of no relationship between number of hits and rate of drumming [*r*(28) = 0.024, *p* = 0.899, BF_10_ = 0.229].

## Discussion

Here we demonstrate the feasibility of using online measurement of infant drumming as an index of infant rhythmic skill. Infants in our sample showed age-appropriate tempo-flexibility, drumming faster to faster tempi and slower to slower tempi. Infants showed evidence of adjusting their rate of drumming away from their intrinsic rate of movement, or Spontaneous Motor Tempo (SMT). However, infants were not close to adult levels of tempo-matching (in the range of tens of milliseconds), showing an average mismatch of greater than 100 ms. This level of tempo-matching is in line with prior observations of 18-month-olds (Rocha and Mareschal, 2017). Previously, 24-month-olds have been shown to synchronise their drumming, but only when interacting with a live, social partner (see Kirschner and Tomasello, 2009; Yu and Myowa, 2021). Whilst our stimuli involved a video recording of a human hand drumming, it was not an overtly “social” signal. Nonetheless, our results show that it is possible to gain a behavioural index of infant sensorimotor synchronisation using a low-cost and accessible open-source platform.

Methodologically this work has multiple strengths. Firstly, by leveraging the Lookit platform, we were able to collect high resolution behavioural data with good ecological validity from a notoriously challenging age group, with minimal experimenter oversight. Because data were collected at home, infants were in a highly familiar setting and caregivers could run the study at time of their choosing. Parents could even abandon an attempt and try at a later time. Secondly, despite the variability of testing circumstances, we were able to get good compliance with instructions and engagement with the task. For example, in several cases older siblings were present but data could be screened for interference and distraction. Finally, the data processing pipeline provided objective measures of movement and rhythmicity with a relatively small amount of manual coding.

Our study demonstrates that markerless motion capture data with infants can be collected in a home with no specialist equipment. The data quality was sufficient for us to extract measures of infant motor tempo with automated Fourier transforms. This is a promising proof of concept, particularly given that the OpenPose model was trained primarily with adult data (Cao et al., 2021). The best infants participants produced data comparable to adult pilot participants (see “Supplementary Material”). It is important to observe that for infants with little or no drumming the Fourier method will not automatically extract a drumming frequency due to lower frequency noise. If additional manual coding was used to tag periods of drumming then more accurate tempo scores could be extracted. Future work will develop this functionality. Further, the trial lengths were purposefully short (20-s), in order to minimise attrition from the study in this unique testing scenario where the experimenter is not present, but this may not have allowed enough time for all infants to provide data. The toddlers mostly tolerated the length very well, and increasing the trial length to 1-min may allow more time for the infants to “warm up” to the drumming and produce enough data for analysis.

One general limitation of this approach to motion capture is that data is only two-dimensional, in the plane of the camera (X and Y coordinates). OpenPose does have the capability to combine data from multiple cameras to reconstruct three-dimensional poses and movement (Nakano et al., 2020). However, this only works in highly optimised conditions with a single participant in view, making it unsuitable for our web-based, parent supervised paradigm. However, the design of data processing workflow allows us to substitute alternative, improved pose estimation algorithms. See Wang et al. (2021) for a review of recent advances.

Future iterations of this paradigm can benefit from clearer instructions to both the caregiver and infant. Of the 68 infants recruited for the study, 18 did not provide drumming data in any of the experimental conditions. Further, the SMT exhibited in the first trial (SMT1) was slower than we would have predicted, and indeed slower than the SMT produced in SMT2, which was recorded at the end of the experiment. The caregivers were asked to prompt the infant “Can you drum for me?”, “Can you show me how you drum?”, but it is possible this was not sufficient for some infants to understand the task. Some parents reported that their child was unfamiliar with the word “drum,” while other infants simply responded “No!”. The original design did not include a video example, in an effort to not bias the infants’ SMT. However, a possible solution would be to include a video montage where two or more infants are drumming side-by-side, giving a clear demonstration of the action expected (repeated whole hand hits), whilst not giving a strong timing signal, adding clarity and motivation to participate. Further, if infants do not produce drumming during SMT1, it could be possible for the parent to replay the demonstration video, and the child attempt the trial again. This would better enable the collection of a representative SMT.

The largest source of missing data was poor camera angles that meant the infant hand could not be tracked (*N* = 14). Lookit is optimised for desktop/laptop computers (i.e., not tablets or mobile phones), and anecdotally, the angle of webcams is normally optimised for centring the adult face. The infant hand is considerably lower in the camera’s field. Prior to commencing the study, the caregiver was shown a preview of their camera angle and asked to check that their infant was in shot. However, as the infant was not yet drumming at this point, it may not have been obvious as to whether the area that would be drummed upon was in view. Future iterations can preview the view of the webcam between each experimental trial, such that the caregiver can adapt their angle as needed. However, care must be taken not to make the task instruction too complex or demanding for caregivers to follow.

A further limitation of remote asynchronous testing that we did not foresee was that not all children were sat at a table that provided good auditory and haptic feedback from the children’s drumming. Subtle differences in the surface infants were drumming on could potentially affect their ability to adapt their behaviour to the auditory stimuli we asked them to synchronise with. Future iterations could therefore also ask the parent and child to “sound check” their drumming to ensure they are getting auditory feedback when they hit the table. Further, if infants are recruited for a study outside of Lookit (e.g., where they are already participating in a lab based session), the paradigm could also be used either within the lab, or at home with a standardised surface (e.g., drum), provided by the research team, to ameliorate these differences.

The study provides promising basis for further exploration of other domains. Firstly, rhythmic movements of the whole body could be examined, allowing investigation of dancing and entrainment to music. OpenPose has been used in this context in laboratory studies with adults (Zeng and Chen, 2021). Our work demonstrates that this could be feasibly done with relatively large samples of young children. Even more promising would be to investigate synchrony between individuals, especially in the context of bonding and responsive caregiving where current human coding measures are labour intensive and lack standardization and predictive validity (Lotzin et al., 2015). Automated solutions have been a goal of social signal processing for a long time (Chetouani et al., 2017) and movement data has emerged as a promising signal (Egmose et al., 2017; López Pérez et al., 2017). However, progress has been slow and most methods are not suitable for field data (Chu et al., 2015). Therefore, we are currently adapting our methods for use in this context.

In summary, here we provide a successful proof-of-concept that we can extract the rate and accuracy of infant drumming from home video, using largely automated and fully open-source procedures. In an initial study of 2-year-old toddlers, we find evidence for tempo-flexibility, but not synchronisation, in response to an isochronous external beat presented at different tempi. The overarching goal of the current work was to develop a tool that is suitable to assess rhythmic movement in very young children, which can be employed at scale, and potentially even identify children at risk of neurodevelopmental disorders, including speech and language difficulties. Such longitudinal assessments, that are appropriate over developmental time, are key to understanding the mechanistic profiles of rhythm impairments across a broad range of neurodevelopmental disorders (Lense et al., 2021). Now that the feasibility of this online approach has been demonstrated, future work can refine the procedure, and further develop this promising tool for deeper insights into infant behaviour.

## Data Availability Statement

The original contributions presented in the study are included in the article/Supplementary Material, further inquiries can be directed to the corresponding author.

## Ethics Statement

The studies involving human participants were reviewed and approved by Goldsmiths, University of London. Verbal informed consent to participate in this study was provided by the participants’ legal guardian via a recorded video statement. Written informed consent was obtained from the minor(s)’ legal guardian, for the publication of any potentially identifiable images or data included in this article.

## Author Contributions

SR and CA co-designed the study, collected and analysed the data, wrote the manuscript, and approved the submitted version.

## Conflict of Interest

The authors declare that the research was conducted in the absence of any commercial or financial relationships that could be construed as a potential conflict of interest.

## Publisher’s Note

All claims expressed in this article are solely those of the authors and do not necessarily represent those of their affiliated organizations, or those of the publisher, the editors and the reviewers. Any product that may be evaluated in this article, or claim that may be made by its manufacturer, is not guaranteed or endorsed by the publisher.

## Footnotes

1
https://lookit.mit.edu
