# Supplementary material for: Assessing Sensorimotor Synchronisation in Toddlers Using the Lookit Online Experiment Platform and Automated Movement Extraction
Source: Front Psychol. 2022 Jun 30;13:897230. doi: 10.3389/fpsyg.2022.897230 (PMC9282044; doi:10.3389/fpsyg.2022.897230)
Supplement: Supplementary file 1 [file Data_Sheet_1.docx]

**Supplementary Materials & Tutorial.**

Found online at <https://github.com/InfantLab/VASC/tree/master/DrumTutorial>

The supplementary materials contain a tutorial helps you walk through using the analysis method with a small subset of the dataset from our paper. It uses the VASC toolkit scripts at the link above to extract rate of drumming from a videos of infants banging on a table. For comparison, two videos of adults performing the same tasks are also included.
